# Occupational stress and burnout among physiotherapists: a cross-sectional survey in Cadiz (Spain)

**DOI:** 10.1186/s12960-020-00537-0

**Published:** 2020-11-25

**Authors:** Ines Carmona-Barrientos, Francisco J. Gala-León, Mercedes Lupiani-Giménez, Alberto Cruz-Barrientos, David Lucena-Anton, Jose A. Moral-Munoz

**Affiliations:** 1 Dept. of Nursing and Physiotherapy, University of Cadiz, Cadiz, Spain; 2 E.U.E. Salus Infirmorum, University of Cadiz, Cadiz, Spain; 3 Institute of Research and Innovation in Biomedical Sciences of the Province of Cadiz (INiBICA), University of Cadiz, Cadiz, Spain

**Keywords:** Physiotherapy, Burnout, Occupational stress

## Abstract

**Background:**

Occupational stress is considered an ongoing epidemic. An inadequate response to a stressful situation can trigger burnout syndrome. In this way, the assistant services (health and teaching) often reach higher levels of burnout. The present study aimed to measure the level of occupational stress and burnout in physiotherapists in the province of Cadiz (Spain), working in the public and/or private sector.

**Methods:**

This was an observational, descriptive and cross-sectional study. A sample of 272 physiotherapists took part in the study. The variables measured were sociodemographic variables, working conditions, level of occupational stress and burnout. Burnout includes three characteristics or dimensions: emotional exhaustion (EE), depersonalization (DP) and personal accomplishment (PA). Correspondence analysis of the sociodemographic, organizational and psychological variables were analyzed using Chi-squared significance tests. Spearman correlations and a linear regression analysis were also carried out to determine the dependence between occupational stress and burnout.

**Results:**

The results showed that 30.51% of physiotherapists suffered from a high level of occupational stress, while 34.56% suffered from an average level. There was a clear dependence between a high level of stress and professionals who felt stressed during their academic training period (*p* = 0.02), those who were in temporary work (*p* = 0.03) and those with over 10 years of professional experience (*p* = 0.05). The overall level of burnout was low, since only the EE dimension had a high value; the average was 21.64 ± 10.57. The DP (6.57 ± 4.65) and PA (39.52 ± 5.97) levels were low. There was a significant dependence (*p* < 0.05) between EE and the following sociodemographic variables: work shift, willingness to study the same degree, stress and inadequate academic training, and a stressful job. In addition, a significant correlation was found between occupational stress and the EE and DP dimensions of burnout.

**Conclusions:**

A high prevalence of occupational stress was detected among physiotherapists in Cadiz (Spain). The levels of occupational stress and its correlation with burnout show that the cumulative effect of stress could lead to burnout. Furthermore, these results regarding occupational stress show the necessity of developing coping strategies for physiotherapists and healthcare staff.

## Background

Sometimes, in a colloquial environment, it is said that a professional is “burned out”, i.e., that a situation (work, family or social) has exhausted his/her capacity to adapt. This situation could become an etiopathogenic factor, because of a continuous process of tension and stress [1]. Burnout syndrome does not have a precise and universally accepted definition. Thus, it alludes to moments of mismatch between the worker and the position he/she occupies, a situation that results in a certain abnormal psychophysiological symptomatology with depressive characteristics [2].

Work, as a human activity, requires a series of contributions: effort, dedication, time, skill, etc. In that way, workers want to perceive not only economic and material compensation, but also psychological and social compensation, which contributes to meeting the needs of human existence [3]. Unfortunately, the reality of work is often different. There are jobs in which performance does not guarantee the worker's safety or with characteristics that do not favor motivation or allow for developing of the worker's self-esteem; in short, they do not offer a good quality of working life [4]. Workers can conceive their tasks as a duty or as a right, and depending on their attitude, they will experience different work-related behaviors [5].

Many work experiences will inevitably influence an individual’s sense of fulfillment and personal satisfaction, but they also affect the worker in the opposite direction. When a worker does not have strategies to face unfavorable work situations or does not have personal resources to adapt, stress appears [6]. Selye, in 1936, developed the concept of stress, defining it as “*a non-specific response of the body to any demand, whether it is caused by, or results in, pleasant or unpleasant conditions*” [7]. This response can have serious and sometimes irreparable consequences for the individual’s health and physical, psychological and social well-being. Occupational stress is highly widespread in our society, because of the interest in productivity and efficiency, not always accompanied by adequate working conditions [8].

From an integrating perspective, we can define occupational stress as the set of emotional, cognitive, physiological and behavioral reactions of a worker to certain harmful aspects of the content, organization, or work environment. If a worker suffers occupational stress and feels he/she cannot cope with the situation [9], there is a mismatch between the individual, the workplace and the organization [10]. This often affects the healthcare providers, patients and organizations [11].

The term “burnout” is used indiscriminately as equivalent to occupational stress. It is necessary to clarify that burnout has its origin in occupational stress, but they are not the same concept. Freudenberger [12] described burnout as a sense of failure and exhaustion in response to an exaggerated demand for personal resources or energy, placing the negative emotions and feelings produced by burnout in a work context [13].

Certain characteristics of health professions could be considered as risk factors, such as many hours at work, clinical conflicts, the outrageous demand of patients who need health care, ethical dilemmas and the fear of lawsuits [14]. Dissatisfaction with work conditions make the individual especially vulnerable to this syndrome [15]. In physiotherapy, personal involvement with patients and family members is high. Moreover, contact is maintained over long periods because of the frequency of interventions in long term or chronic pathologies [16]. Based on scientific results [17], burnout could be higher in physiotherapists than in other health professions. In a national survey, the results of 172 physiotherapists from the private and public sectors were analyzed; the results showed that approximately 57% who worked in the public sector and 40% who worked in the private sector affirmed that their work was stressful [18]. In this sense, the sociodemographic characteristics that may influence the origin of stress are not well described.

According to the literature, occupational stress affects health professionals to a considerable degree. Some studies have measured burnout in physicians [19], nurses [20] and other health professions [21]. Regarding physiotherapy, articles about burnout are scarce compared to other health professions [1, 22–26]. Furthermore, there have also been few studies performed in Spain with good methodological quality and a representative sample [16, 27–29].

Therefore, regarding this background, the aim of this study was to determine the prevalence of occupational stress and burnout in physiotherapists in the province of Cadiz (Spain). The levels of involvement in both pathologies and their dimensions were analyzed. Some sociodemographic characteristics that may have an influence as triggers of occupational stress and/or burnout are reported.

## Methods

The present study was a descriptive, cross-sectional and correlational study. Two validated questionnaires were used to analyze the level of occupational stress by the “Escala de estrés socio laboral” (EAE-S) (Socio-occupational stress scale) [30], and burnout by the Maslach Burnout Inventory (MBI) [31].

### Sample and procedures

Respondents for this study were physiotherapists working in the province of Cadiz (Spain) during February–April 2016 in the public or private sector, or both. We obtained the census of collegiate physiotherapists for the province of Cadiz from the professional council of Andalusia (Spain), the “Ilustre Colegio de Fisioterapia de Andalucía”, with a total of 750 collegiate professionals. The list of professionals working in the public sector was obtained from the Andalusian health service, the “Servicio Andaluz de Salud”. Furthermore, the center web pages were searched on the Internet to reach the largest number of physiotherapists working in the private sector. A total of 404 active workers was determined. Telephone calls and e-mails were sent to staff coordinators, asking them to distribute our survey to physiotherapists. If they agreed to take part, the surveys were delivered and collected in paper format in a single session by the main author of the present manuscript (I.C.B.). The study was conducted under the ethical standards laid down by the Helsinki Declaration [32]. All the participants read and signed the informed consent; participation was voluntary, and the survey was completed anonymously.

Physiotherapists between 20 and 65 years of active working age at the time of data collection were included. We excluded physiotherapists who were out of work or unemployed. Although we wanted to survey the total of active workers (n = 404), the collegiate professionals census (n = 750) was selected to the sample size calculation, in order to get more accurate information. The following formula for calculating the sample size to estimate a proportion with finite population was used: $$n = \frac{{{\text{N*}}Z_{\alpha }^{2} p*\left( {1 - p} \right)}}{{d^{2} *\left( {N - 1} \right) + Z_{\alpha }^{2} *p*\left( {1 - p} \right)}}$$, where n is the sample size with finite population correction, N is the population size, $${Z}_{\alpha }^{2}$$ is the Z statistic for a level of confidence (1.962 for 95% confidence interval), *p* is the expected proportion (0.05 in our case), and *d* is the precision. We obtained a representative sample with confidence interval α = 0.05 and d = 0.05, with at least 265 respondents.

### Measures

#### Sociodemographic and self-constructed questions

This questionnaire collected sociodemographic data, such as gender, age, marital status, number of children, professional experience, employment sector, type of contract, management tasks and work timetable/shift. Furthermore, some self-constructed questions were added to assess the worker’s perception about the training received at university and current occupational stress.

#### Occupational stress scale

This scale focused on people of working age in the range of 20 to 60 years of age. It comprises 50 items distributed in three areas of context: work characteristics, work context and relationship of the individual with work. Each item reports on: 1) the presence or not of different stressful events in the work context (Yes or No); 2) the time when the event took place (N if it occurs at present or P if it occurred in the past); and 3) a personal assessment of the intensity with which these events have had an effect (assessed with a rating scale from 0 to 3 points).

The results were established from the total intensity scores according to the centile values using a percentile criterion (33%), assuming that the upper-, middle- and lower-third of the sample express high, medium and low levels of occupational stress [30].

#### Maslach Burnout Inventory

The version of the MBI used has 22 items distributed in three dimensions: emotional exhaustion (EE) (9 items), depersonalization (DP) (5 items), and personal accomplishment (PA) (8 items). EE refers to a sense physical and mental exhaustion, entailing a lack of energy for daily work [12]. DP is defined as a set of negative feelings, causing isolation, coldness and even cynicism in daily interactions with coworkers [33]. The lack of PA alludes to a negative self-image and dissatisfaction with work, which is detrimental to the person receiving the service. The items are evaluated on a frequency rating scale of seven levels: never (0), a few times a year or less (1), once a month or less (2), a few times a month (3), once a week (4), a few times a week (5) and every day (6). The MBI is created specifically for professions that involve human treatment or service, considering sanitary and teaching professions as the closest approximation of the test.

High scores on the subscales of EE and DP and low scores on the PA subscale indicate high burnout [31].

### Statistical analysis

The statistical package Statgraphics 18.0.8 for Windows (Manugistics Inc., Rockville, MD, USA) and the Microsoft Office 365 package were used for data processing and statistical analysis. The profile of the sample was obtained through direct scores (absolute values) and percentages (relative values) of the sociodemographic questionnaire data. No method for dealing with missing data were used. The following statistical analyses were also performed to obtain study results: (a) the Kolmogorov–Smirnov test was used to check the normal distribution of the sample; (b) correspondence analysis of the sociodemographic, organizational and psychological variables, was performed through Chi-squared significance tests; (c) Spearman correlations and a linear regression analysis were also performed.

## Results

From the total of 404 physiotherapists, 272 active workers (67.33%) took part in the study (103 men and 169 women). Missing data were found in sociodemographic characteristics, but not in the EAE-S and MBI. In this sample, 62% were female, 79.04% were between 26 and 50 years of age, 63.10% worked in the private sector, 22.14% worked in the public sector and 14.76% worked in both. Table 1 shows information about the sociodemographic profile and the self-constructed questions of the surveyed professionals. Additional data show the dependence analysis of the sociodemographic variables through Chi-squared significance tests (see Additional file 1). The test was calculated for the 13 variables. When the test was significant, this indicated a relationship between the two analyzed variables.

**Table 1:** Sociodemographic and self-constructed questions

Item	Variables	MD	No.	%
1	Gender (*n* = 272)			
	Male	0	103	37.87
	Female		169	62.13
2	Age (n = 272)			
	< 25 years	0	43	15.81
	26–50 years		215	79.04
	> 50 years		14	5.15
3	Marital status (*n* = 272)			
	Single	0	146	53.68
	Married		115	42.28
	Divorced/separated		11	4.04
4	Number of children (*n* = 272)			
	0	0	165	60.66
	1		39	14.34
	2+		68	25.00
5	Management tasks (*n* = 270)			
	Yes	2	133	49.26
	No		137	50.74
6	Willingness to study the same degree (*n* = 268)			
	Yes	4	225	83.96
	No		43	16.04
7	Stress during training at university (*n* = 246)			
	No	26	132	53.66
	Some		83	33.74
	Quite		31	12.60
8	Employment sector (*n* = 271)			
	Public	1	60	22.14
	Private		171	63.10
	Both		40	14.76
9	Type of contract (*n* = 270)			
	Permanent	2	166	61.48
	Acting worker		18	6.67
	Temporary		86	31.85
10	Professional experience (n = 272)			
	0–2 years	0	37	13.60
	3–10 years		107	39.34
	> 10 years		128	47.06
11	Timetable/shift (*n* = 272)			
	Morning	0	71	26.10
	Afternoon		33	12.13
	Morning and afternoon		145	53.31
	Rotating		23	8.46
12	Adequate training at university (*n* = 271)			
	Yes	1	74	27.31
	No		197	72.69
13	My job is stressful (*n* = 246)			
	No	26	47	19.11
	Some		120	48.78
	Quite		79	32.11

*MD* missing data

According to the results obtained in the EAE-S, 30.51% of physiotherapists suffered from a high level of stress, 34.56% experienced a moderate level, 27.94% had a low level and 6.99% had no stress. Regarding the relationship between the sociodemographic variables and the level of occupational stress (Table 2), there could be a relationship between female gender and higher levels of stress. However, this relationship was not statistically significant. Some self-constructed questions are highly discriminative regarding the level of stress. Thus, the professionals who felt stressed during their academic training period continued to experience the highest levels of stress (*p* = 0.02). There was also a clear dependence between high level of stress in physiotherapists who were in temporary work (*p* = 0.03). The variable years of experience was nearly significant at *p* = 0.05, so professionals with over 10 years of professional experience suffered more stress.

**Table 2:** Sociodemographic and self-constructed questions associated with occupational stress

Item	Variable	Category	Level of occupational stress, *n* (%)*					
			No	Low	Moderate	High	*χ*^2^	*p*
1	Gender	Male	11 (10.7)	33 (32.0)	34 (33.0)	25 (24.3)	6.47	0.09
		Female	8 (4.7)	43 (25.5)	60 (35.5)	58 (34.3)		
7	Stress during training at university	No	10 (7.6)	44 (33.3)	48 (36.3)	30 (22.7)	14.75	0.02**
		Some	4 (4.8)	17 (20.5)	23 (27.7)	39 (47.0)		
		Quite	3 (9.7)	8 (25.8)	11 (35.5)	9 (29.0)		
9	Type of contract	Permanent	12 (7.23)	56 (33.73)	57 (34.34)	41 (24.70)	13.47	0.03**
		Acting worker	1 (5.56)	1 (5.56)	9 (50.00)	7 (38.89)		
		Temporary	6 (7.0)	18 (20.9)	27 (31.4)	35 (40.7)		
10	Professional experience	0–2 years	5 (13.5)	7 (18.9)	18 (48.7)	7 (18.9)	12.34	0.05
		3–10 years	4 (3.8)	27 (25.2)	40 (37.4)	36 (33.6)		
		> 10 years	10 (7.8)	42 (32.8)	36 (28.1)	40 (31.3)		

* Percentages are of total respondents in each variable category (% of row totals)

** Significant (*p* < 0.05)

Only values *p* < 0.10 are presented

Regarding the three dimensions of burnout measured with the MBI, we can state that the level of this pathology was low. Only the EE dimension had a high score, with a mean value of 21.64 ± 10.57, showing an average EE level. The DP (6.57 ± 4.65) and PA (39.52 ± 5.97) levels were low. Tables 3, 4, 5 show the relationship between the sociodemographic variables and the three dimensions of the MBI.

**Table 3:** Sociodemographic and self-constructed questions associated with emotional exhaustion

Item	Variable	Category	Emotional exhaustion, *n* (%)*				
			Low	Moderate	High	*χ*^2^	*p*
6	Willingness to study the same degree	Yes	75 (33.5)	93 (41.5)	56 (25.0)	24.23	0.00**
		No	6 (13.9)	10 (23.2)	27 (62.8)		
7	Stress during training at university	No	51 (38.9)	53 (40.4)	27 (20.6)	39.48	0.00**
		Some	15 (18.0)	37 (44.6)	31 (37.3)		
		Quite	4 (12.9)	4 (12.9)	23 (74.2)		
11	Timetable/shift	Morning	27 (38.5)	26 (37.1)	17 (24.3)	13.26	0.04**
		Afternoon	16 (48.5)	9 (27.2)	8 (24.2)		
		Morning and afternoon	33 (22.7)	59 (40.7)	53 (36.5)		
		Rotating	5 (21.7)	11 (47.8)	7 (30.4)		
12	Adequate training at university	Yes	30 (41.1)	25 (34.2)	18 (24.6)	6.06	0.04**
		No	51 (25.9)	79 (40.1)	67 (34.0)		
13	My job is stressful	No	26 (56.2)	15 (32.6)	5 (10.8)	44.14	0.00^**^
		Some	33 (27.5)	56 (46.6)	31 (25.8)		
		Quite	11 (13.5)	25 (30.8)	45 (55.5)		

* Percentages are of total respondents in each variable category (% of row totals)

** Significant (*p* < 0.05)

Only values *p* < 0.10 are presented

**Table 4:** Sociodemographic and self-constructed questions associated with depersonalization

Item	Variable	Category	Depersonalization, *n* (%)*				
			Low	Moderate	High	*χ*^2^	*p*
10	Professional experience	0–2 years	9 (25.0)	13 (36.1)	14 (38.9)	8.36	0.09
		3–10 years	27 (25.2)	30 (28.0)	50 (46.7)		
		> 10 years	44 (34.4)	47 (36.7)	37 (28.9)		
11	Timetable/shift	Morning	28 (40.0)	20 (28.5)	22 (31.4)	11.56	0.07
		Afternoon	10 (30.3)	10 (30.3)	13 (39.4)		
		Morning and afternoon	41 (28.2)	51 (35.1)	53 (36.5)		
		Rotating	1 (4.3)	9 (39.1)	13 (56.5)		
13	My job is stressful	No	23 (50.0)	13 (28.2)	10 (21.7)	17.98	0.00**
		Some	35 (29.1)	42 (35.0)	43 (35.8)		
		Quite	14 (17.3)	26 (32.1)	41 (50.6)		

* Percentages are of total respondents in each variable category (% of row totals)

** Significant (*p* < 0.05)

Only values *p* < 0.10 are presented

**Table 5:** Sociodemographic and self-constructed questions associated with personal accomplishment

Item	Variable	Category	Personal accomplishment, *n* (%)*				
			Low	Moderate	High	χ^2^	*p*
5	Management tasks	Yes	27 (20.0)	55 (40.7)	53 (39.2)	15.23	0.00**
		No	57 (41.9)	41 (30.1)	38 (27.9)		
8	Employment sector	Public	27 (45.0)	18 (30.0)	15 (25.0)	8.05	0.09
		Private	49 (28.6)	62 (26.2)	60 (35.1)		
		Both	8 (20.5)	16 (41.0)	15 (38.4)		

* Percentages are of total respondents in each variable category (% of row totals)

** Significant (*p* < 0.05)

Only values *p* < 0.10 are presented

The variables significantly dependent on EE are the work shift from the sociodemographic data and those related to the worker’s perceptions of training and actual occupational stress (those who think their training at university was stressful, the training was not adequate, the profession is also stressful and would not study the same degree again). In the DP dimension, physiotherapy was seen as a stressful profession had a significant dependence. In PA, there was a very high dependence (with a *p*-value of 0.0005) among professionals who perform management tasks.

**Fig. 1:**
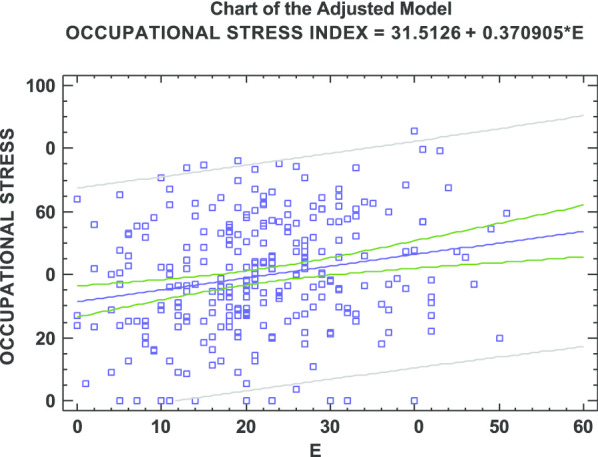
Scatter plot of occupational stress vs. emotional exhaustion

**Fig. 2:**
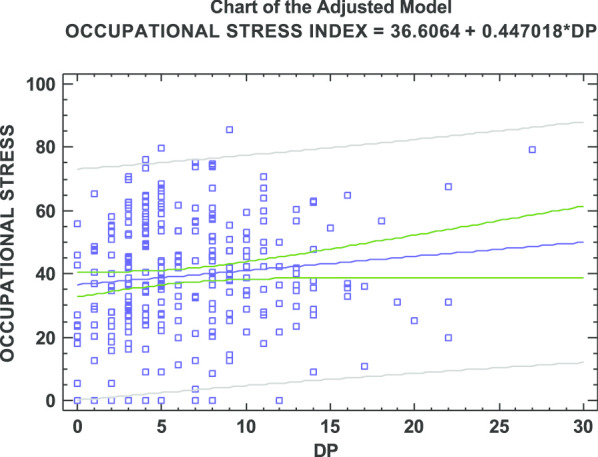
Scatter plot of occupational stress vs. depersonalization

Finally, a significant correlation was found between occupational stress and the EE and DP dimensions of burnout, as shown in Figs. 1 and 2. A linear regression model is proposed in which the response variable is the level of work stress and the independent variables are EE and DP, respectively. In this way, although there was a dependence (*p* = 0.0004) between occupational stress and EE, a low correlation was observed. The coefficient of determination (R^2^ = 0.0449) showed that only 4.49% of the variability of stress was explained by EE. Regarding DP, since *p* = 0.06, a linear independence between both variables could be accepted. DP, however, explained only 1.26% of the stress variable (R^2^ = 0.0126).

## Discussion

The main aim of this study was to determine the levels of occupational stress and burnout and the relationship with sociodemographic variables in a sample of physiotherapists. Regarding the sample profile, 62% of the participants were female. This percentage is similar to that reported by the World Confederation for Physical Therapy (WCPT) [34] in Spain. Most of them were in the age range between 26 and 50 years, in permanent work, with over 10 years of professional experience, providing their services in the private sector and working in the morning and afternoon. Two-thirds of our sample suffered medium to high levels of occupational stress. Professionals whose work was temporary or those with over 10 years of experience showed a significant relationship with the highest levels of occupational stress. Although the burnout index was low (measured in its three dimensions), the values for the EE dimension were worrisome, since one-third of the participants had a high or moderately high EE level. This dimension showed a statistically significant dependence with the morning and afternoon or rotating shift. Likewise, there was a relationship between EE and physiotherapists who perceived their training as inadequate or who considered the profession stressful. In the literature, similar profiles could be found in terms of gender, marital status, age, years of experience, etc. [24, 27, 35].

Regarding the moderately high level of stress we observed, we consider that this value could be alarming, taking into account that stress could trigger burnout syndrome [36, 37]. In a doctoral dissertation in which the sample included physiotherapists working in the public sector in Cadiz (Spain), the moderately high stress values were around 30% of the total [38]. In our study, the number of participants was more than double, so the trend seems to increase. Moreover, in a study in which different health professions were evaluated (nurses, clinical assistants, physicians and physiotherapists), physiotherapists had the highest levels of stress [39]. Santos et al. [29] obtained similar results in terms of moderate levels of stress, although with a lower percentage of high stress.

According to the sociodemographic characteristics, females suffered the highest levels of high and moderate occupational stress, although this was not significant. Contrarily, men scored slightly higher at the level of mild stress and the absence of stress. We did not find significant differences in stress between gender in the literature [16, 29, 40, 41]. Nevertheless, in other studies analyzing pathologies associated with stress [42], episodes of anxiety [43], taking psychotropic drugs [44] and psychopathology [44, 45], females presented higher rates than men. In that sense, it is important to note that the concept of occupational stress is exactly identified with burnout in some studies. In our study, the prevalence of both concepts was evaluated separately, so when trying to contrast our results with published studies, only data about burnout were found.

The dependence found between high stress and temporality in the workplace is not surprising, since the security guaranteed by having a permanent job undoubtedly contributes to a state of psychosocial well-being [1, 8]. The years of professional experience were also a determinant since workers suffering from the highest values of stress had less than 2 and over 10 years of experience. In the first case, it is because of the stress of inexperience and insecurity with various treatment skills and/or in the relationship with their patients. In the second case, stress levels are higher because of the accumulation of psychological strain by praxis and other vital events, more related to the sense of burnout. These results are contradictory to those found in the research literature. Śliwiński et al. in 2014 observed that professionals with over 15 years of experience felt more professional satisfaction [46]; this variable was described as protective or buffering of burnout [15, 22, 47].

The results showed that the physiotherapists who perceived their studies as stressful during their training period had higher levels of stress at work. This finding highlights the importance that the personality might have on the perception of stress (locus of control, coping ability, tolerance to frustration, etc.) [48].

As stated before, the number of studies comparing and reporting on occupational stress and burnout in physiotherapists is low; however, we found that the values obtained in each dimension of burnout were very similar to different studies in other professions [16, 23, 25, 28, 49]. Gender, age, marital status, years of experience, work shift and performing management tasks were the most commonly studied sociodemographic characteristics. Similar to other studies, we did not find significant differences for gender or age [18], although there was a certain association between EE and female gender [50] and DP and male gender [18, 51]. Regarding age, there was an association between high EE and middle-aged participants. This is worrisome because it could increase in the future to burnout syndrome if nothing is done to prevent it. The middle-aged population was determined to be the most predisposed to suffer from higher levels of burnout [52]. A split work shift (morning and afternoon) had a significant relationship with the EE and a rotating shift with DP. It is not well-defined as to which work shift predisposes one to the development of burnout syndrome, but some authors agree that the number of hours spent at work has a cumulative influence on this syndrome [16, 53].

As stated at the beginning of the discussion, there was a remarkably strong correlation found between EE and DP with some self-constructed questions. These variables alluded to seeing physiotherapy as a stressful profession. It seems obvious, considering that occupational stress is the precursor of burnout [37, 54–57].

Finally, we should also highlight that the results show a very high interrelation between professionals who perform management tasks and high PA. There are also contradictory findings on this point, as some articles support the notion that management gives some autonomy to a professional that could help with preventing or coping with burnout [58]; other authors stated that adding excessive bureaucratization and administrative paperwork to the daily workload could reinforce the development of burnout [59]. Even in a study analyzing the relationship between occupational stress and burnout in professionals performing only management tasks, the results were ambivalent [17]. Professional satisfaction could have a protective or cushioning effect on coping with burnout [46]. Different actions aimed at preventing and mitigating the effects of burnout need to take this issue into account [47].

The high levels of occupational stress obtained could show that we are facing a population of physiotherapists with a certain risk of suffering burnout. To prevent this, information about coping strategies for stress is essential. These strategies should apply at the individual level, at the organizational level and in the individual–organization relationship [22]. In this way, we should focus actions on the stressor elements and the resources available to the worker, taking into account that the same stressor creates different subjective experiences in each person.

Although the present study presents some relevant findings, some limitations should be addressed. First, it is necessary to point out the limited research documents that have analyzed occupational stress and burnout in physiotherapists. For this reason, we needed to discuss our findings concerning articles related to other health professions. Moreover, the self-administered survey and the volume of questions on the scales included in our study could have introduced a bias since subjects could have answered the questions randomly. Finally, it is clear that we require a longitudinal approach to analyze the evolution of psychopathologies studied in a specific period.

## Conclusions

A high prevalence of occupational stress was detected among physiotherapists in Cadiz (Spain), as more than half of the sample suffered from a moderate to high level of occupational stress. The prevalence of burnout syndrome in our sample was low. Concerning the EE dimension, more than a third of the sample suffered from moderately high levels. The least significant dimension in burnout was PA.

Occupational stress was higher in females and professionals with temporary contracts. There was also a relationship between stress and workers with over 10 years of experience (indicating that the effects of occupational stress are cumulative).

Concerning the EE dimension, physiotherapists under 25 and over 50 years of age and working in the morning and afternoon had the highest scores. If we associate the self-constructed questions about the subjects’ perception about the stressful aspects of the profession and academic training, the highest levels of EE were obtained from those who thought their training was stressful, the training was not adequate, the profession was stressful and would not study the same degree again. Regarding PA, the professionals who had the highest levels were those who worked on a rotating shift, had between 3 and 10 years of experience and perceived the profession as stressful. The performance of management tasks correlated with perceiving greater PA.

This overview brings to light the necessity of developing coping strategies for occupational stress and burnout for physiotherapists. On the one hand, this state of stress could have a cumulative effect on the professional and lead to burnout, considered an established pathology. On the other hand, it is important to notice the implications that this could have on the patient. Physiotherapy, similar to other health professions, involves constant and direct treatment with the patient, so if the DP associated with burnout increases, it could negatively affect the patient. This drawback should be our priority to solve due for clear ethical reasons. Certainly, a follow-up study is needed, including the same population to study the evolution of stress and to determine if those at risk of burnout eventually experienced it.

Some findings make us question the cultural or sociodemographic aspects that could influence developing occupational stress and burnout. Attending to the current scientific literature, there have been few studies carried out in the physiotherapist population and even fewer longitudinal studies to allow us to measure the cumulative effect of stress and its relationship with burnout. In view of this situation, well-conducted longitudinal studies are needed. Nevertheless, the results obtained here could provide a research-based overview with which the health committee should set up organizational guidelines. These organizational efforts could increase job satisfaction among physiotherapists and help prevent occupational stress and burnout.

## Supplementary information

**Additional file 1:** Additional tables.

## Data Availability

The datasets generated and/or analyzed during the current study are not publicly available due to ethical issues related to participant confidentiality.

## Abbreviations

EAE-S: Socio-occupational stress scale
MBI: Maslach Burnout Inventory
EE: Emotional exhaustion
DP: Depersonalization
PA: Personal accomplishment
WCPT: World Confederation for Physical Therapy
