# Supplementary material for: Occupational stress and burnout among physiotherapists: a cross-sectional survey in Cadiz (Spain)
Source: Hum Resour Health. 2020 Nov 25;18:91. doi: 10.1186/s12960-020-00537-0 (PMC7690107; doi:10.1186/s12960-020-00537-0)
Supplement: Supplementary file 1 — Additional file 1: Additional tables. [file 12960_2020_537_MOESM1_ESM.docx]

**Supplementary material**

| ***Item*** | ***Variables*** | | ***Gender, n(%)^*^*** | | ***χ^2^*** | ***p*** |
| --- | --- | --- | --- | --- | --- | --- |
|  |  | | **Male** | ***Female*** |  |  |
| **1.** | **Gender** | Male | - | - | - | - |
|  |  | Female | - | - |  |  |
| **2.** | **Age** | <25 yrs | 15 (5.51) | 28 (10.29) | 4.43 | 0.10 |
|  |  | 26 - 50 yrs | 79 (29.04) | 136 (50) |  |  |
|  |  | >50 yrs | 9 (3.31) | 5 (1.84) |  |  |
| **3.** | **Marital status** | Single | 53 (1949) | 93 (34.19) | 1.11 | 0.57 |
|  |  | Married | 47 (17.28) | 68 (25) |  |  |
|  |  | Divorced/Separated | 3 (1.1) | 8 (2.94) |  |  |
| **4.** | **Number of children** | 0 | 60 (22.06) | 105 (38.6) | 0.88 | 0.64 |
|  |  | 1 | 14 (5.15) | 25 (9.19) |  |  |
|  |  | 2+ | 29 (10.66) | 39 (14.34) |  |  |
| **5.** | **Management tasks** | Yes | 59 (21.69) | 76 (27.94) | 3.88 | 0.04^**^ |
|  |  | No | 44 (16.18) | 93 (34.19) |  |  |
| **6.** | **Willingness to study the same degree** | Yes | 86 (32.09) | 138 (51.49) | 0.63 | 0.72 |
|  |  | No | 16 (5.97) | 27 (10.07) |  |  |
| **7.** | **Stress during the training at University** | No | 55 (22.36) | 77 (31.3) | 3.13 | 0.37 |
|  |  | Some | 28 (11.38) | 55 (22.36) |  |  |
|  |  | Quite | 13 (5.28) | 17 (6.91) |  |  |
| **8.** | **Employment sector** | Public | 24 (8.86) | 36 (13.28) | 5.25 | 0.07 |
|  |  | Private | 57 (21.03) | 114 (42.07) |  |  |
|  |  | Both | 21 (7.75) | 19 (7.01) |  |  |
| **9.** | **Type of contract** | Permanent | 70 (25.93) | 96 (35.56) | 3.78 | 0.15 |
|  |  | Acting worker | 4 (1.48) | 14 (5.19) |  |  |
|  |  | Temporary | 29 (10.74) | 57 (21.11) |  |  |
| **10.** | **Professional experience** | 0-2 yrs | 15 (5.51) | 22 (8.09) | 3.77 | 0.15 |
|  |  | 3 - 10 yrs | 33 (12.13) | 74 (27.21) |  |  |
|  |  | >10 yrs | 55 (20.22) | 73 (26.84) |  |  |
| **11.** | **Timetable/shift** | Morning | 28 (10.29) | 43 (15.81) | 7.22 | 0.06 |
|  |  | Afternoon | 9 (3.31) | 24 (8.82) |  |  |
|  |  | Morning and afternoon | 62 (22.79) | 83 (30.51) |  |  |
|  |  | Rotating | 4 (1.47) | 19 (6.99) |  |  |
| **12.** | **Adequate training at University** | Yes | 30 (11.07) | 44 (16.24) | 0.27 | 0.59 |
|  |  | No | 73 (26.94) | 124 (45.76) |  |  |
| **13.** | **My job is stressful** | No | 24 (9.68) | 23 (9.27) | 5.30 | 0.07 |
|  |  | Some | 39 (15.73) | 81 (32.66) |  |  |
|  |  | Quite | 34 (13.71) | 47 (18.95) |  |  |
| ^*^ Percentages are of total respondents in each variable category (% of row totals).  ^**^ Significant (p<0.05)  *χ^2^: Chi-square value*  *p: p-value* | | | | | | |

Table S1. Variables associated with Gender.

Table S2. Variables associated with Age.

| ***Item*** | ***Variables*** | | ***Age, n(%)^*^*** | | | ***χ^2^*** | ***p*** |
| --- | --- | --- | --- | --- | --- | --- | --- |
|  |  | | **<25** | ***26 - 50*** | ***>50*** |  |  |
| **1.** | **Gender** | Male | 15 (5.51) | 79 (29.04) | 9 (3.31) | 4.43 | 0.10 |
|  |  | Female | 28 (10.29) | 136 (50) | 5 (1.84) |  |  |
| **2.** | **Age** | <25 yrs | - | - | - | - | - |
|  |  | 26 - 50 yrs | - | - | - |  |  |
|  |  | >50 yrs | - | - | - |  |  |
| **3.** | **Marital status** | Single | 41 (15.07) | 105 (38.6) | 0 (0) | 48.41 | 0.00^**^ |
|  |  | Married | 2 (0.74) | 100 (36.76) | 13 (4.78) |  |  |
|  |  | Divorced/Separated | 0 (0) | 10 (3.68) | 1 (0.37) |  |  |
| **4.** | **Number of children** | 0 | 42 (15.44) | 123 (45.22) | 0 (0) | 48.02 | 0.00^**^ |
|  |  | 1 | 0 (0) | 33 (12.13) | 6 (2.21) |  |  |
|  |  | 2+ | 1 (0.37) | 59 (21.69) | 8 (2.94) |  |  |
| **5.** | **Management tasks** | Yes | 19 (6.99) | 109 (40.07) | 7 (2.57) | 0.60 | 0.73 |
|  |  | No | 24 (8.82) | 106 (38.97) | 7 (2.57) |  |  |
| **6.** | **Willingness to study the same degree** | Yes | 37 (13.81) | 177 (66.04) | 10 (3.73) | 2.43 | 0.65 |
|  |  | No | 5 (1.87) | 34 (12.69) | 4 (1.49) |  |  |
| **7.** | **Stress during the training at University** | No | 18 (7.32) | 108 (43.9) | 6 (2.44) | 8.92 | 0.17 |
|  |  | Some | 11 (4.47) | 69 (28.05) | 3 (1.22) |  |  |
|  |  | Quite | 10 (4.07) | 18 (7.32) | 2 (0.81) |  |  |
| **8.** | **Employment sector** | Public | 2 (0.74) | 50 (18.45) | 8 (2.95) | 20.01 | 0.00^**^ |
|  |  | Private | 33 (12.18) | 135 (49.82) | 3 (1.11) |  |  |
|  |  | Both | 8 (2.95) | 29 (10.7) | 3 (1.11) |  |  |
| **9.** | **Type of contract** | Permanent | 8 (2.96) | 145 (53.7) | 13 (4.81) | 43.75 | 0.00^**^ |
|  |  | Acting worker | 5 (1.85) | 13 (4.81) | 0 (0) |  |  |
|  |  | Temporary | 30 (11.11) | 55 (20.37) | 1 (0.37) |  |  |
| **10.** | **Professional experience** | 0-2 yrs | 27 (9.93) | 10 (3.68) | 0 (0) | 121.59 | 0.00^**^ |
|  |  | 3 - 10 yrs | 15 (5.51) | 91 (33.46) | 1 (0.37) |  |  |
|  |  | >10 yrs | 1 (0.37) | 114 (41.91) | 13 (4.78) |  |  |
| **11.** | **Timetable/shift** | Morning | 3 (1.1) | 58 (21.32) | 10 (3.68) | 32.22 | 0.00^**^ |
|  |  | Afternoon | 11 (4.04) | 22 (8.09) | 0 (0) |  |  |
|  |  | Morning and afternoon | 22 (8.09) | 119 (43.75) | 4 (1.47) |  |  |
|  |  | Rotating | 7 (2.57) | 16 (5.88) | 0 (5.15) |  |  |
| **12.** | **Adequate training at University** | Yes | 18 (6.64) | 53 (19.56) | 3 (1.11) | 5.46 | 0.06 |
|  |  | No | 25 (9.23) | 162 (59.78) | 10 (3.69) |  |  |
| **13.** | **My job is stressful** | No | 2 (0.81) | 39 (15.73) | 6 (2.42) | 20.62 | 0.00^**^ |
|  |  | Some | 16 (6.45) | 102 (41.13) | 2 (0.81) |  |  |
|  |  | Quite | 21 (8.47) | 57 (22.98) | 3 (1.21) |  |  |
| ^*^ Percentages are of total respondents in each variable category (% of row totals).  ^**^ Significant (p<0.05)  *χ^2^: Chi-square value*  *p: p-value* | | | | | | | |

Table S3. Variables associated with Marital status.

| ***Item*** | ***Variables*** | | ***Marital status, n(%)^*^*** | | | ***χ^2^*** | ***p*** |
| --- | --- | --- | --- | --- | --- | --- | --- |
|  |  | | **Single** | ***Married*** | ***Divorced/Separated*** |  |  |
| **1.** | **Gender** | Male | 53 (19.49) | 47 (17.28) | 3 (1.1) | 1.11 | 0.57 |
|  |  | Female | 93 (34.19) | 68 (25) | 8 (2.94) |  |  |
| **2.** | **Age** | <25 yrs | 41 (15.07) | 2 (0.74) | 0 (0) | 48.41 | 0.00^**^ |
|  |  | 26 - 50 yrs | 105 (38.6) | 100 (36.76) | 10 (3.68) |  |  |
|  |  | >50 yrs | 0 (0) | 13 (4.78) | 1 (0.37) |  |  |
| **3.** | **Marital status** | Single | - | - | - | - | - |
|  |  | Married | - | - | - |  |  |
|  |  | Divorced/Separated | - | - | - |  |  |
| **4.** | **Number of children** | 0 | 1 (51.84) | 2 (7.72) | 3 (1.1) | 172.45 | 0.00^**^ |
|  |  | 1 | 3 (1.1) | 32 (11.76) | 4 (1.47) |  |  |
|  |  | 2+ | 2 (0.74) | 62 (22.79) | 4 (1.47) |  |  |
| **5.** | **Management tasks** | Yes | 68 (25) | 62 (22.79) | 5 (1.84) | 1.46 | 0.48 |
|  |  | No | 78 (28.68) | 53 (19.49) | 6 (2.21) |  |  |
| **6.** | **Willingness to study the same degree** | Yes | 122 (45.52) | 93 (34.7) | 9 (3.36) | 1.79 | 0.77 |
|  |  | No | 20 (7.46) | 21 (7.84) | 2 (0.75) |  |  |
| **7.** | **Stress during the training at University** | No | 69 (28.05) | 57 (23.17) | 6 (2.44) | 3.62 | 0.72 |
|  |  | Some | 44 (17.89) | 36 (14.63) | 3 (1.22) |  |  |
|  |  | Quite | 20 (8.13) | 9 (3.66) | 1 (0.41) |  |  |
| **8.** | **Employment sector** | Public | 18 (6.64) | 39 (14.39) | 3 (1.11) | 18.66 | 0.00^**^ |
|  |  | Private | 106 (39.11) | 59 (21.77) | 6 (2.21) |  |  |
|  |  | Both | 22 (8.12) | 16 (5.9) | 2 (0.74) |  |  |
| **9.** | **Type of contract** | Permanent | 71 (26.3) | 86 (31.85) | 9 (3.33) | 23.65 | 0.00^**^ |
|  |  | Acting worker | 10 (3.7) | 8 (2.96) | 0 (0) |  |  |
|  |  | Temporary | 64 (23.7) | 20 (7.41) | 2 (0.74) |  |  |
| **10.** | **Professional experience** | 0 - 2 yrs | 36 (13.24) | 1 (0.37) | 0 (0) | 103.08 | 0.00^**^ |
|  |  | 3 - 10 yrs | 82 (30.15) | 23 (8.46) | 2 (0.74) |  |  |
|  |  | >10 yrs | 28 (10.29) | 91 (33.46) | 9 (3.31) |  |  |
| **11.** | **Timetable/shift** | Morning | 22 (8.09) | 45 (16.54) | 4 (1.47) | 22.61 | 0.00^**^ |
|  |  | Afternoon | 22 (8.09) | 10 (3.68) | 1 (0.37) |  |  |
|  |  | Morning and afternoon | 85 (31.25) | 54 (19.85) | 6 (2.21) |  |  |
|  |  | Rotating | 17 (6.25) | 6 (2.21) | 0 (0) |  |  |
| **12.** | **Adequate training at University** | Yes | 53 (19.49) | 47 (17.28) | 3 (1.1) | 4.54 | 0.10 |
|  |  | No | 93 (34.19) | 68 (25) | 8 (2.94) |  |  |
| **13.** | **My job is stressful** | No | 41 (15.07) | 2 (0.74) | 0 (0) | 9.02 | 0.06 |
|  |  | Some | 105 (38.6) | 100 (36.76) | 10 (3.68) |  |  |
|  |  | Quite | 0 (0) | 13 (4.78) | 1 (0.37) |  |  |
| ^*^ Percentages are of total respondents in each variable category (% of row totals).  ^**^ Significant (p<0.05)  *χ^2^: Chi-square value*  *p: p-value* | | | | | | | |

Table S4. Variables associated with Number of children.

| ***Item*** | ***Variables*** | | ***Number of children, n(%)^*^*** | | | ***χ^2^*** | ***p*** |
| --- | --- | --- | --- | --- | --- | --- | --- |
|  |  | | **0** | ***1*** | ***>2*** |  |  |
| **1.** | **Gender** | Male | 60 (22.06) | 14 (5.15) | 29 (10.66) | 0.88 | 0.64 |
|  |  | Female | 105 (38.6) | 25 (9.19) | 39 (14.34) |  |  |
| **2.** | **Age** | <25 yrs | 42 (15.44) | 0 (0) | 1 (0.37) | 48.02 | 0.00^**^ |
|  |  | 26 - 50 yrs | 123 (45.22) | 33 (12.13) | 59 (21.69) |  |  |
|  |  | >50 yrs | 0 (0) | 6 (2.21) | 8 (2.94) |  |  |
| **3.** | **Marital status** | Single | 141 (51.84) | 3 (1.1) | 2 (0.74) | 172.45 | 0.00^**^ |
|  |  | Married | 21 (7.72) | 32 (11.76) | 62 (22.79) |  |  |
|  |  | Divorced/Separated | 3 (1.1) | 4 (1.47) | 4 (1.47) |  |  |
| **4.** | **Number of children** | 0 | - | - | - | - | - |
|  |  | 1 | - | - | - |  |  |
|  |  | 2+ | - | - | - |  |  |
| **5.** | **Management tasks** | Yes | 79 (29.04) | 21 (7.72) | 35 (12.87) | 0.57 | 0.75 |
|  |  | No | 86 (31.62) | 18 (6.62) | 33 (12.13) |  |  |
| **6.** | **Willingness to study the same degree** | Yes | 24 (51.12) | 6 (11.94) | 13 (20.52) | 1.28 | 0.86 |
|  |  | No | 137 (8.96) | 32 (2.24) | 55 (4.85) |  |  |
| **7.** | **Stress during the training at University** | No | 75 (30.49) | 18 (7.32) | 39 (15.85) | 7.37 | 0.28 |
|  |  | Some | 54 (21.95) | 11 (4.47) | 18 (7.32) |  |  |
|  |  | Quite | 22 (8.94) | 4 (1.63) | 4 (1.63) |  |  |
| **8.** | **Employment sector** | Public | 23 (8.49) | 10 (3.69) | 27 (9.96) | 21.47 | 0.28 |
|  |  | Private | 118 (43.54) | 24 (8.86) | 29 (10.7) |  |  |
|  |  | Both | 24 (8.86) | 5 (1.85) | 11 (4.06) |  |  |
| **9.** | **Type of contract** | Permanent | 84 (31.11) | 29 (10.74) | 53 (61.48) | 23.42 | 0.00^**^ |
|  |  | Acting worker | 10 (3.7) | 2 (0.74) | 6 (6.67) |  |  |
|  |  | Temporary | 70 (25.93) | 7 (2.59) | 9 (31.85) |  |  |
| **10.** | **Professional experience** | 0-2 yrs | 36 (13.24) | 1 (0.37) | 0 (13.6) | 99.38 | 0.00^**^ |
|  |  | 3 - 10 yrs | 90 (33.09) | 11 (4.04) | 6 (39.34) |  |  |
|  |  | >10 yrs | 39 (14.34) | 27 (9.93) | 62 (47.06) |  |  |
| **11.** | **Timetable/shift** | Morning | 24 (8.82) | 18 (6.62) | 29 (10.66) | 34.72 | 0.00^**^ |
|  |  | Afternoon | 26 (9.56) | 3 (1.1) | 4 (1.47) |  |  |
|  |  | Morning and afternoon | 97 (35.66) | 14 (5.15) | 34 (12.5) |  |  |
|  |  | Rotating | 18 (6.62) | 4 (1.47) | 1 (0.37) |  |  |
| **12.** | **Adequate training at University** | Yes | 50 (18.45) | 9 (3.32) | 15 (5.54) | 1.94 | 0.37 |
|  |  | No | 115 (42.44) | 29 (10.7) | 53 (19.56) |  |  |
| **13.** | **My job is stressful** | No | 21 (8.47) | 4 (1.61) | 22 (8.87) | 20.12 | 0.00^**^ |
|  |  | Some | 70 (28.23) | 21 (8.47) | 29 (11.69) |  |  |
|  |  | Quite | 61 (24.6) | 8 (3.23) | 12 (4.84) |  |  |
| ^*^ Percentages are of total respondents in each variable category (% of row totals).  ^**^ Significant (p<0.05)  *χ^2^: Chi-square value*  *p: p-value* | | | | | | | |

Table S5. Variables associated with performing management task.

| ***Item*** | ***Variables*** | | ***Management tasks, n(%)^*^*** | | ***χ^2^*** | ***p*** |
| --- | --- | --- | --- | --- | --- | --- |
|  |  | | **Yes** | ***No*** |  |  |
| **1.** | **Gender** | Male | 59 (21.69) | 44 (16.18) | 3.88 | 0.04^**^ |
|  |  | Female | 76 (27.94) | 93 (34.19) |  |  |
| **2.** | **Age** | <25 yrs | 19 (6.99) | 24 (8.82) | 0.60 | 0.73 |
|  |  | 26 - 50 yrs | 109 (40.07) | 106 (38.97) |  |  |
|  |  | >50 yrs | 7 (2.57) | 7 (2.57) |  |  |
| **3.** | **Marital status** | Single | 68 (25) | 78 (28.68) | 1.46 | 0.48 |
|  |  | Married | 62 (22.79) | 53 (19.49) |  |  |
|  |  | Divorced/Separated | 5 (1.84) | 6 (2.21) |  |  |
| **4.** | **Number of children** | 0 | 79 (29.04) | 86 (31.62) | 0.57 | 0.75 |
|  |  | 1 | 21 (7.72) | 18 (6.62) |  |  |
|  |  | 2+ | 35 (12.87) | 33 (12.13) |  |  |
| **5.** | **Management tasks** | Yes | - | - | - | - |
|  |  | No | - | - |  |  |
| **6.** | **Willingness to study the same degree** | Yes | 119 (44.4) | 105 (39.18) | 10.21 | 0.00^**^ |
|  |  | No | 12 (4.48) | 31 (11.57) |  |  |
| **7.** | **Stress during the training at University** | No | 70 (28.46) | 62 (25.2) | 2.57 | 0.46 |
|  |  | Some | 37 (15.04) | 46 (18.7) |  |  |
|  |  | Quite | 16 (6.5) | 14 (5.69) |  |  |
| **8.** | **Employment sector** | Public | 19 (7.01) | 41 (15.13) | 10.14 | 0.00^**^ |
|  |  | Private | 95 (35.06) | 76 (28.04) |  |  |
|  |  | Both | 20 (7.38) | 20 (7.38) |  |  |
| **9.** | **Type of contract** | Permanent | 96 (35.56) | 70 (25.93) | 12.76 | 0.00^**^ |
|  |  | Acting worker | 7 (2.59) | 11 (4.07) |  |  |
|  |  | Temporary | 30 (11.11) | 56 (20.74) |  |  |
| **10.** | **Professional experience** | 0-2 yrs | 13 (4.78) | 24 (8.82) | 3.74 | 0.15 |
|  |  | 3 - 10 yrs | 57 (20.96) | 50 (18.38) |  |  |
|  |  | >10 yrs | 65 (23.9) | 63 (23.16) |  |  |
| **11.** | **Timetable/shift** | Morning | 28 (10.29) | 43 (15.81) | 12.63 | 0.00^**^ |
|  |  | Afternoon | 10 (3.68) | 23 (8.46) |  |  |
|  |  | Morning and afternoon | 85 (31.25) | 60 (22.06) |  |  |
|  |  | Rotating | 12 (4.41) | 11 (4.04) |  |  |
| **12.** | **Adequate training at University** | Yes | 39 (1439) | 35 (1292) | 0.33 | 0.56 |
|  |  | No | 96 (3542) | 101 (3727) |  |  |
| **13.** | **My job is stressful** | No | 25 (10.08) | 22 (8.87) | 0.22 | 0.89 |
|  |  | Some | 59 (23.79) | 61 (24.6) |  |  |
|  |  | Quite | 41 (16.53) | 40 (16.13) |  |  |
| ^*^ Percentages are of total respondents in each variable category (% of row totals).  ^**^ Significant (p<0.05)  *χ^2^: Chi-square value*  *p: p-value* | | | | | | |

Table S6. Variables associated with the willing of study the same degree.

| ***Item*** | ***Variables*** | | ***Willingness to study the same degree, n(%)^*^*** | | ***χ^2^*** | ***p*** |
| --- | --- | --- | --- | --- | --- | --- |
|  |  | | **Yes** | ***No*** |  |  |
| **1.** | **Gender** | Male | 1 (32.09) | 0 (5.97) | 0.63 | 0.72 |
|  |  | Female | 138 (51.49) | 27 (10.07) |  |  |
| **2.** | **Age** | <25 yrs | 37 (13.81) | 5 (1.87) | 2.43 | 0.65 |
|  |  | 26 - 50 yrs | 177 (66.04) | 34 (12.69) |  |  |
|  |  | >50 yrs | 10 (3.73) | 4 (1.49) |  |  |
| **3.** | **Marital status** | Single | 122 (45.52) | 20 (7.46) | 1.79 | 0.77 |
|  |  | Married | 93 (34.7) | 21 (7.84) |  |  |
|  |  | Divorced/Separated | 9 (3.36) | 2 (0.75) |  |  |
| **4.** | **Number of children** | 0 | 137 (51.12) | 24 (8.96) | 1.28 | 0.86 |
|  |  | 1 | 32 (11.94) | 6 (2.24) |  |  |
|  |  | 2+ | 55 (20.52) | 13 (4.85) |  |  |
| **5.** | **Management tasks** | Yes | 119 (44.4) | 12 (4.48) | 10.21 | 0.00^**^ |
|  |  | No | 105 (39.18) | 31 (11.57) |  |  |
| **6.** | **Willingness to study the same degree** | Yes | - | - | - | - |
|  |  | No | - | - |  |  |
| **7.** | **Stress during the training at University** | No | 115 (46.94) | 17 (6.94) | 10.13 | 0.11 |
|  |  | Some | 68 (27.76) | 14 (5.71) |  |  |
|  |  | Quite | 19 (7.76) | 10 (4.08) |  |  |
| **8.** | **Employment sector** | Public | 44 (16.48) | 16 (5.99) | 7.20 | 0.12 |
|  |  | Private | 144 (53.93) | 23 (8.61) |  |  |
|  |  | Both | 35 (13.11) | 4 (1.5) |  |  |
| **9.** | **Type of contract** | Permanent | 137 (51.5) | 25 (9.4) | 1.48 | 0.83 |
|  |  | Acting worker | 16 (6.02) | 2 (0.75) |  |  |
|  |  | Temporary | 69 (25.94) | 16 (6.02) |  |  |
| **10.** | **Professional experience** | 0-2 yrs | 31 (11.57) | 5 (1.87) | 5.34 | 0.25 |
|  |  | 3 - 10 yrs | 95 (35.45) | 12 (4.48) |  |  |
|  |  | >10 yrs | 98 (36.57) | 26 (9.7) |  |  |
| **11.** | **Timetable/shift** | Morning | 57 (21.27) | 14 (5.22) | 4.31 | 0.63 |
|  |  | Afternoon | 28 (10.45) | 4 (1.49) |  |  |
|  |  | Morning and afternoon | 122 (45.52) | 19 (7.09) |  |  |
|  |  | Rotating | 17 (6.34) | 6 (2.24) |  |  |
| **12.** | **Adequate training at University** | Yes | 67 (25.09) | 6 (2.25) | 5.07 | 0.07 |
|  |  | No | 156 (58.43) | 37 (13.86) |  |  |
| **13.** | **My job is stressful** | No | 40 (16.26) | 7 (2.85) | 10.09 | 0.03^**^ |
|  |  | Some | 105 (42.68) | 13 (5.28) |  |  |
|  |  | Quite | 59 (23.98) | 21 (8.54) |  |  |
| ^*^ Percentages are of total respondents in each variable category (% of row totals).  ^**^ Significant (p<0.05)  *χ^2^: Chi-square value*  *p: p-value* | | | | | | |

Table S7. Variables associated with the sensation of feeling missed because of their studies.

| ***Item*** | ***Variables*** | | ***Stress during the training at University, n(%)^*^*** | | | ***χ^2^*** | ***p*** |
| --- | --- | --- | --- | --- | --- | --- | --- |
|  |  | | **No** | ***Some*** | ***Quite*** |  |  |
| **1.** | **Gender** | Male | 55 (22.36) | 28 (11.38) | 13 (5.28) | 3.88 | 0.04^**^ |
|  |  | Female | 77 (31.3) | 55 (22.36) | 17 (6.91) |  |  |
| **2.** | **Age** | <25 yrs | 18 (7.32) | 11 (4.47) | 10 (4.07) | 8.92 | 0.17 |
|  |  | 26 - 50 yrs | 108 (43.9) | 69 (28.05) | 18 (7.32) |  |  |
|  |  | >50 yrs | 6 (2.44) | 3 (1.22) | 2 (0.81) |  |  |
| **3.** | **Marital status** | Single | 69 (28.05) | 44 (17.89) | 20 (8.13) | 7.37 | 0.28 |
|  |  | Married | 57 (23.17) | 36 (14.63) | 9 (3.66) |  |  |
|  |  | Divorced/Separated | 6 (2.44) | 3 (1.22) | 1 (0.41) |  |  |
| **4.** | **Number of children** | 0 | 75 (30.49) | 54 (21.95) | 22 (8.94) | 3.62 | 0.72 |
|  |  | 1 | 18 (7.32) | 11 (4.47) | 4 (1.63) |  |  |
|  |  | 2+ | 39 (15.85) | 18 (7.32) | 4 (1.63) |  |  |
| **5.** | **Management tasks** | Yes | 70 (28.46) | 37 (15.04) | 16 (6.5) | 2.57 | 0.46 |
|  |  | No | 62 (25.2) | 46 (18.7) | 14 (5.69) |  |  |
| **6.** | **Willingness to study the same degree** | Yes | 115 (46.94) | 68 (27.76) | 19 (7.76) | 10.13 | 0.11 |
|  |  | No | 17 (6.94) | 14 (5.71) | 10 (4.08) |  |  |
| **7.** | **Stress during the training at University** | No | - | - | - | - | - |
|  |  | Some | - | - | - |  |  |
|  |  | Quite | - | - | - |  |  |
| **8.** | **Employment sector** | Public | 22 (8.98) | 22 (8.98) | 4 (1.63) | 9.28 | 0.15 |
|  |  | Private | 86 (35.1) | 50 (20.41) | 21 (8.57) |  |  |
|  |  | Both | 23 (9.39) | 11 (4.49) | 5 (2.04) |  |  |
| **9.** | **Type of contract** | Permanent | 87 (35.51) | 46 (18.78) | 14 (5.71) | 21.01 | 0.00^**^ |
|  |  | Acting worker | 7 (2.86) | 8 (3.27) | 1 (0.41) |  |  |
|  |  | Temporary | 37 (15.1) | 29 (11.84) | 15 (6.12) |  |  |
| **10.** | **Professional experience** | 0-2 yrs | 18 (7.32) | 8 (3.25) | 7 (2.85) | 5.83 | 0.44 |
|  |  | 3 - 10 yrs | 50 (20.33) | 38 (15.45) | 12 (4.88) |  |  |
|  |  | >10 yrs | 64 (26.02) | 37 (15.04) | 11 (4.47) |  |  |
| **11.** | **Timetable/shift** | Morning | 38 (15.45) | 18 (7.32) | 2 (0.81) | 10.72 | 0.29 |
|  |  | Afternoon | 14 (5.69) | 11 (4.47) | 2 (0.81) |  |  |
|  |  | Morning and afternoon | 68 (27.64) | 46 (18.7) | 24 (9.76) |  |  |
|  |  | Rotating | 12 (4.88) | 8 (3.25) | 2 (0.81) |  |  |
| **12.** | **Adequate training at University** | Yes | 36 (14.63) | 24 (9.76) | 5 (10.16) | 2.14 | 0.54 |
|  |  | No | 96 (39.02) | 59 (23.98) | 25 (2.03) |  |  |
| **13.** | **My job is stressful** | No | 42 (17.14) | 5 (2.04) | 0 (0) | 71.05 | 0.00^**^ |
|  |  | Some | 66 (26.94) | 48 (19.59) | 4 (1.63) |  |  |
|  |  | Quite | 24 (9.8) | 29 (11.84) | 26 (10.61) |  |  |
| ^*^ Percentages are of total respondents in each variable category (% of row totals).  ^**^ Significant (p<0.05)  *χ^2^: Chi-square value*  *p: p-value* | | | | | | | |

Table S8. Variables associated with Employment sector.

| ***Item*** | ***Variables*** | | ***Employment sector, n(%)^*^*** | | | ***χ^2^*** | ***p*** |
| --- | --- | --- | --- | --- | --- | --- | --- |
|  |  | | **Public** | ***Private*** | ***Both*** |  |  |
| **1.** | **Gender** | Male | 24 (8.86) | 57 (21.03) | 21 (7.75) | 5.25 | 0.07 |
|  |  | Female | 36 (13.28) | 114 (42.07) | 19 (7.01) |  |  |
| **2.** | **Age** | <25 yrs | 2 (0.74) | 33 (12.18) | 8 (2.95) | 20.01 | 0.00^**^ |
|  |  | 26 - 50 yrs | 50 (18.45) | 135 (49.82) | 29 (10.7) |  |  |
|  |  | >50 yrs | 8 (2.95) | 3 (1.11) | 3 (1.11) |  |  |
| **3.** | **Marital status** | Single | 18 (6.64) | 106 (39.11) | 22 (8.12) | 18.66 | 0.00^**^ |
|  |  | Married | 39 (14.39) | 59 (21.77) | 16 (5.9) |  |  |
|  |  | Divorced/Separated | 3 (1.11) | 6 (2.21) | 2 (0.74) |  |  |
| **4.** | **Number of children** | 0 | 23 (8.49) | 118 (43.54) | 24 (8.86) | 21.47 | 0.00^**^ |
|  |  | 1 | 10 (3.69) | 24 (8.86) | 5 (1.85) |  |  |
|  |  | 2+ | 27 (9.96) | 29 (10.7) | 11 (4.06) |  |  |
| **5.** | **Management tasks** | Yes | 19 (7.01) | 95 (35.06) | 20 (7.38) | 10.14 | 0.00^**^ |
|  |  | No | 41 (15.13) | 76 (28.04) | 20 (7.38) |  |  |
| **6.** | **Willingness to study the same degree** | Yes | 44 (16.48) | 144 (53.93) | 35 (13.11) | 7.20 | 0.12 |
|  |  | No | 16 (5.99) | 23 (8.61) | 4 (1.5) |  |  |
| **7.** | **Stress during the training at University** | No | 22 (8.98) | 86 (35.1) | 23 (9.39) | 9.28 | 0.15 |
|  |  | Some | 22 (8.98) | 50 (20.41) | 11 (4.49) |  |  |
|  |  | Quite | 4 (1.63) | 21 (8.57) | 5 (2.04) |  |  |
| **8.** | **Employment sector** | Public | - | - | - | - | - |
|  |  | Private | - | - | - |  |  |
|  |  | Both | - | - | - |  |  |
| **9.** | **Type of contract** | Permanent | 40 (14.87) | 109 (40.52) | 16 (5.95) | 16.77 | 0.00^**^ |
|  |  | Acting worker | 8 (2.97) | 8 (2.97) | 2 (0.74) |  |  |
|  |  | Temporary | 12 (4.46) | 53 (19.7) | 21 (7.81) |  |  |
| **10.** | **Professional experience** | 0-2 yrs | 1 (0.37) | 29 (10.7) | 7 (2.58) | 56.08 | 0.00^**^ |
|  |  | 3 - 10 yrs | 7 (2.58) | 88 (32.47) | 12 (4.43) |  |  |
|  |  | >10 yrs | 52 (19.19) | 54 (19.93) | 21 (7.75) |  |  |
| **11.** | **Timetable/shift** | Morning | 36 (13.28) | 26 (9.59) | 9 (3.32) | 50.94 | 0.00^**^ |
|  |  | Afternoon | 6 (2.21) | 25 (9.23) | 2 (0.74) |  |  |
|  |  | Morning and afternoon | 14 (5.17) | 106 (39.11) | 24 (8.86) |  |  |
|  |  | Rotating | 4 (1.48) | 14 (5.17) | 5 (1.85) |  |  |
| **12.** | **Adequate training at University** | Yes | 14 (5.19) | 48 (17.78) | 11 (4.07) | 0.42 | 0.80 |
|  |  | No | 45 (16.67) | 123 (45.56) | 29 (10.74) |  |  |
| **13.** | **My job is stressful** | No | 11 (4.45) | 24 (9.72) | 12 (4.86) | 5.48 | 0.24 |
|  |  | Some | 22 (8.91) | 79 (31.98) | 18 (7.29) |  |  |
|  |  | Quite | 15 (6.07) | 56 (22.67) | 10 (4.05) |  |  |
| ^*^ Percentages are of total respondents in each variable category (% of row totals).  ^**^ Significant (p<0.05)  *χ^2^: Chi-square value*  *p: p-value* | | | | | | | |

Table S9. Variables associated with Type of contract.

| ***Item*** | ***Variables*** | | ***Type of contract, n(%)^*^*** | | | ***χ^2^*** | ***P*** |
| --- | --- | --- | --- | --- | --- | --- | --- |
|  |  | | **Permanent** | ***Acting worker*** | ***Temporary*** |  |  |
| **1.** | **Gender** | Male | 70 (25.93) | 4 (1.48) | 29 (10.74) | 3.78 | 0.15 |
|  |  | Female | 96 (35.56) | 14 (5.19) | 57 (21.11) |  |  |
| **2.** | **Age** | <25 yrs | 8 (2.96) | 5 (1.85) | 30 (11.11) | 43.75 | 0.00^**^ |
|  |  | 26 - 50 yrs | 145 (53.7) | 13 (4.81) | 55 (20.37) |  |  |
|  |  | >50 yrs | 13 (4.81) | 0 (0) | 1 (0.37) |  |  |
| **3.** | **Marital status** | Single | 71 (26.3) | 10 (3.7) | 64 (23.7) | 26.65 | 0.00^**^ |
|  |  | Married | 86 (31.85) | 8 (2.96) | 20 (7.41) |  |  |
|  |  | Divorced/Separated | 9 (3.33) | 0 (0) | 2 (0.74) |  |  |
| **4.** | **Number of children** | 0 | 84 (31.11) | 10 (3.7) | 70 (25.93) | 23.42 | 0.00^**^ |
|  |  | 1 | 29 (10.74) | 2 (0.74) | 7 (2.59) |  |  |
|  |  | 2+ | 53 (19.63) | 6 (2.22) | 9 (3.33) |  |  |
| **5.** | **Management tasks** | Yes | 96 (35.56) | 7 (2.59) | 30 (11.11) | 12.76 | 0.00^**^ |
|  |  | No | 70 (25.93) | 11 (4.07) | 56 (20.74) |  |  |
| **6.** | **Willingness to study the same degree** | Yes | 137 (51.5) | 16 (6.02) | 69 (6.02) | 1.48 | 0.83 |
|  |  | No | 25 (9.4) | 2 (0.75) | 16 (0.75) |  |  |
| **7.** | **Stress during the training at University** | No | 87 (35.51) | 7 (2.86) | 37 (53.47) | 21.01 | 0.00^**^ |
|  |  | Some | 46 (18.78) | 8 (3.27) | 29 (33.88) |  |  |
|  |  | Quite | 14 (5.71) | 1 (0.41) | 15 (12.24) |  |  |
| **8.** | **Employment sector** | Public | 40 (14.87) | 8 (2.97) | 12 (4.46) | 16.77 | 0.00^**^ |
|  |  | Private | 109 (40.52) | 8 (2.97) | 53 (19.7) |  |  |
|  |  | Both | 16 (5.95) | 2 (0.74) | 21 (7.81) |  |  |
| **9.** | **Type of contract** | Permanent | - | - | - | - | - |
|  |  | Acting worker | - | - | - |  |  |
|  |  | Temporary | - | - | - |  |  |
| **10.** | **Professional experience** | 0-2 yrs | 6 (2.22) | 3 (1.11) | 28 (10.37) | 43.95 | 0.00^**^ |
|  |  | 3 - 10 yrs | 66 (24.44) | 7 (2.59) | 33 (12.22) |  |  |
|  |  | >10 yrs | 94 (34.81) | 8 (2.96) | 25 (9.26) |  |  |
| **11.** | **Timetable/shift** | Morning | 56 (20.74) | 5 (1.85) | 10 (3.7) | 46.63 | 0.00^**^ |
|  |  | Afternoon | 9 (3.33) | 3 (1.11) | 20 (7.41) |  |  |
|  |  | Morning and afternoon | 95 (35.19) | 10 (3.7) | 39 (14.44) |  |  |
|  |  | Rotating | 6 (2.22) | 0 (0) | 17 (6.3) |  |  |
| **12.** | **Adequate training at University** | Yes | 36 (13.38) | 5 (1.86) | 32 (11.9) | 6.77 | 0.03^**^ |
|  |  | No | 129 (47.96) | 13 (4.83) | 54 (20.07) |  |  |
| **13.** | **My job is stressful** | No | 31 (12.55) | 2 (0.81) | 13 (5.26) | 9.31 | 0.05 |
|  |  | Some | 78 (31.58) | 11 (4.45) | 31 (12.55) |  |  |
|  |  | Quite | 41 (16.6) | 4 (1.62) | 36 (14.57) |  |  |
| ^*^ Percentages are of total respondents in each variable category (% of row totals).  ^**^ Significant (p<0.05)  *χ^2^: Chi-square value*  *p: p-value* | | | | | | | |

Table S10. Variables associated with Professional experience.

| ***Item*** | ***Variables*** | | ***Professional experience, n(%)^*^*** | | | ***χ^2^*** | ***p*** |
| --- | --- | --- | --- | --- | --- | --- | --- |
|  |  | | **0 - 2** | ***3 - 10*** | ***>10*** |  |  |
| **1.** | **Gender** | Male | 15 (5.51) | 33 (12.13) | 55 (20.22) | 3.77 | 0.15 |
|  |  | Female | 22 (8.09) | 74 (27.21) | 73 (26.84) |  |  |
| **2.** | **Age** | <25 yrs | 27 (9.93) | 15 (5.51) | 1 (0.37) | 121.59 | 0.00^**^ |
|  |  | 26 - 50 yrs | 10 (3.68) | 91 (33.46) | 114 (41.91) |  |  |
|  |  | >50 yrs | 0 (0) | 1 (0.37) | 13 (4.78) |  |  |
| **3.** | **Marital status** | Single | 36 (13.24) | 82 (30.15) | 28 (10.29) | 103.08 | 0.00^**^ |
|  |  | Married | 1 (0.37) | 23 (8.46) | 91 (33.46) |  |  |
|  |  | Divorced/Separated | 0 (0) | 2 (0.74) | 9 (3.31) |  |  |
| **4.** | **Number of children** | 0 | 36 (13.24) | 90 (33.09) | 39 (14.34) | 99.38 | 0.00^**^ |
|  |  | 1 | 1 (0.37) | 11 (4.04) | 27 (9.93) |  |  |
|  |  | 2+ | 0 (0) | 6 (2.21) | 62 (22.79) |  |  |
| **5.** | **Management tasks** | Yes | 13 (4.78) | 57 (20.96) | 65 (23.9) | 3.74 | 0.15 |
|  |  | No | 24 (8.82) | 50 (18.38) | 63 (23.16) |  |  |
| **6.** | **Willingness to study the same degree** | Yes | 31 (11.57) | 95 (35.45) | 98 (36.57) | 5.34 | 0.25 |
|  |  | No | 5 (1.87) | 12 (4.48) | 26 (9.7) |  |  |
| **7.** | **Stress during the training at University** | No | 18 (7.32) | 50 (20.33) | 64 (26.02) | 5.83 | 8.44 |
|  |  | Some | 8 (3.25) | 38 (15.45) | 37 (15.04) |  |  |
|  |  | Quite | 7 (2.85) | 12 (4.88) | 11 (4.47) |  |  |
| **8.** | **Employment sector** | Public | 1 (0.37) | 7 (2.58) | 52 (19.19) | 53.08 | 0.00^**^ |
|  |  | Private | 29 (10.7) | 88 (32.47) | 54 (19.93) |  |  |
|  |  | Both | 7 (2.58) | 12 (4.43) | 21 (7.75) |  |  |
| **9.** | **Type of contract** | Permanent | 6 (2.22) | 66 (24.44) | 94 (34.81) | 43.95 | 0.00^**^ |
|  |  | Acting worker | 3 (1.11) | 7 (2.59) | 8 (2.96) |  |  |
|  |  | Temporary | 28 (10.37) | 33 (12.22) | 25 (9.26) |  |  |
| **10.** | **Professional experience** | 0-2 yrs | - | - | - | - | - |
|  |  | 3 - 10 yrs | - | - | - |  |  |
|  |  | >10 yrs | - | - | - |  |  |
| **11.** | **Timetable/shift** | Morning | 4 (1.47) | 16 (5.88) | 51 (18.75) | 34.14 | 0.00^**^ |
|  |  | Afternoon | 11 (4.04) | 13 (4.78) | 9 (3.31) |  |  |
|  |  | Morning and afternoon | 18 (6.62) | 67 (24.63) | 60 (22.06) |  |  |
|  |  | Rotating | 4 (1.47) | 11 (4.04) | 8 (2.94) |  |  |
| **12.** | **Adequate training at University** | Yes | 17 (6.27) | 31 (11.44) | 26 (9.59) | 9.61 | 0.15 |
|  |  | No | 20 (7.38) | 76 (28.04) | 101 (37.27) |  |  |
| **13.** | **My job is stressful** | No | 4 (1.61) | 11 (4.44) | 32 (12.9) | 14.44 | 0.00^**^ |
|  |  | Some | 14 (5.65) | 52 (20.97) | 54 (21.77) |  |  |
|  |  | Quite | 15 (6.05) | 38 (15.32) | 28 (11.29) |  |  |
| ^*^ Percentages are of total respondents in each variable category (% of row totals).  ^**^ Significant (p<0.05)  *χ^2^: Chi-square value*  *p: p-value* | | | | | | | |

Table S11. Variables associated with Timetable/shift.

| ***Item*** | ***Variables*** | | ***Timetable/shift, n(%)^*^*** | | | | ***χ^2^*** | ***p*** |
| --- | --- | --- | --- | --- | --- | --- | --- | --- |
|  |  | | **Morning** | ***Afternoon*** | ***Morning and Afternoon*** | ***Rotating*** |  |  |
| **1.** | **Gender** | Male | 28 (10.29) | 9 (3.31) | 62 (22.79) | 4 (1.47) | 7.22 | 0.06 |
|  |  | Female | 43 (15.81) | 24 (8.82) | 83 (30.51) | 19 (6.99) |  |  |
| **2.** | **Age** | <25 yrs | 3 (1.1) | 11 (4.04) | 22 (8.09) | 7 (2.57) | 32.22 | 0.00^**^ |
|  |  | 26 - 50 yrs | 58 (21.32) | 22 (8.09) | 119 (43.75) | 16 (5.88) |  |  |
|  |  | >50 yrs | 10 (3.68) | 0 (0) | 4 (1.47) | 0 (0) |  |  |
| **3.** | **Marital status** | Single | 22 (8.09) | 22 (8.09) | 85 (31.25) | 17 (6.25) | 22.61 | 0.00^**^ |
|  |  | Married | 45 (16.54) | 10 (3.68) | 54 (19.85) | 6 (2.21) |  |  |
|  |  | Divorced/Separated | 4 (1.47) | 1 (0.37) | 6 (2.21) | 0 (0) |  |  |
| **4.** | **Number of children** | 0 | 24 (8.82) | 26 (9.56) | 97 (35.66) | 18 (6.62) | 34.72 | 0.00^**^ |
|  |  | 1 | 18 (6.62) | 3 (1.1) | 14 (5.15) | 4 (1.47) |  |  |
|  |  | 2+ | 29 (10.66) | 4 (1.47) | 34 (12.5) | 1 (0.37) |  |  |
| **5.** | **Management tasks** | Yes | 28 (10.29) | 10 (3.68) | 85 (31.25) | 12 (4.41) | 12.63 | 0.00^**^ |
|  |  | No | 43 (15.81) | 23 (8.46) | 60 (22.06) | 11 (4.04) |  |  |
| **6.** | **Willingness to study the same degree** | Yes | 57 (21.27) | 28 (10.45) | 122 (45.52) | 17 (6.34) | 4.31 | 0.63 |
|  |  | No | 14 (5.22) | 4 (1.49) | 19 (7.09) | 6 (2.24) |  |  |
| **7.** | **Stress during the training at University** | No | 38 (15.45) | 14 (5.69) | 68 (27.64) | 12 (4.88) | 10.72 | 0.29 |
|  |  | Some | 18 (7.32) | 11 (4.47) | 46 (18.7) | 8 (3.25) |  |  |
|  |  | Quite | 2 (0.81) | 2 (0.81) | 24 (9.76) | 2 (0.81) |  |  |
| **8.** | **Employment sector** | Public | 36 (13.28) | 6 (2.21) | 14 (5.17) | 4 (1.48) | 50.94 | 0.00^**^ |
|  |  | Private | 26 (9.59) | 25 (9.23) | 106 (39.11) | 14 (5.17) |  |  |
|  |  | Both | 9 (3.32) | 2 (0.74) | 24 (8.86) | 5 (1.85) |  |  |
| **9.** | **Type of contract** | Permanent | 56 (20.74) | 9 (3.33) | 95 (35.19) | 6 (2.22) | 46.63 | 0.00^**^ |
|  |  | Acting worker | 5 (1.85) | 3 (1.11) | 10 (3.7) | 0 (0) |  |  |
|  |  | Temporary | 10 (3.7) | 20 (7.41) | 39 (14.44) | 17 (6.3) |  |  |
| **10.** | **Professional experience** | 0-2 yrs | 4 (1.47) | 11 (4.04) | 18 (6.62) | 4 (1.47) | 34.14 | 0.00^**^ |
|  |  | 3 - 10 yrs | 16 (5.88) | 13 (4.78) | 67 (24.63) | 11 (4.04) |  |  |
|  |  | >10 yrs | 51 (18.75) | 9 (3.31) | 60 (22.06) | 8 (2.94) |  |  |
| **11.** | **Timetable/shift** | Morning | - | - | - | - | - | - |
|  |  | Afternoon | - | - | - | - |  |  |
|  |  | Morning and afternoon | - | - | - | - |  |  |
|  |  | Rotating | - | - | - | - |  |  |
| **12.** | **Adequate training at University** | Yes | 15 (5.54) | 13 (4.8) | 37 (13.65) | 9 (3.32) | 5.50 | 0.13 |
|  |  | No | 55 (20.3) | 20 (7.38) | 108 (39.85) | 14 (5.17) |  |  |
| **13.** | **My job is stressful** | No | 17 (6.85) | 6 (2.42) | 22 (8.87) | 2 (0.81) | 7.67 | 0.26 |
|  |  | Some | 27 (10.89) | 10 (4.03) | 70 (28.23) | 13 (5.24) |  |  |
|  |  | Quite | 15 (6.05) | 10 (4.03) | 48 (19.35) | 8 (3.23) |  |  |
| ^*^ Percentages are of total respondents in each variable category (% of row totals).  ^**^ Significant (p<0.05)  *χ^2^: Chi-square value*  *p: p-value* | | | | | | | | |

Table S12. Variables associated with the thought about the training received during the degree.

| ***Item*** | ***Variables*** | | **Adequate training at University*, n(%)^*^*** | | ***χ^2^*** | ***p*** |
| --- | --- | --- | --- | --- | --- | --- |
|  |  | | **Yes** | ***No*** |  |  |
| **1.** | **Gender** | Male | 30 (11.07) | 73 (26.94) | 0.27 | 0.59 |
|  |  | Female | 44 (16.24) | 124 (45.76) |  |  |
| **2.** | **Age** | <25 yrs | 18 (6.64) | 25 (9.23) | 5.46 | 0.06 |
|  |  | 26 - 50 yrs | 53 (19.56) | 162 (59.78) |  |  |
|  |  | >50 yrs | 3 (1.11) | 10 (3.69) |  |  |
| **3.** | **Marital status** | Single | 47 (17.34) | 99 (36.53) | 4.54 | 0.10 |
|  |  | Married | 26 (9.59) | 89 (32.84) |  |  |
|  |  | Divorced/Separated | 1 (0.37) | 9 (3.32) |  |  |
| **4.** | **Number of children** | 0 | 50 (18.45) | 115 (42.44) | 1.94 | 0.37 |
|  |  | 1 | 9 (3.32) | 29 (10.7) |  |  |
|  |  | 2+ | 15 (5.54) | 53 (19.56) |  |  |
| **5.** | **Management tasks** | Yes | 39 (14.39) | 96 (35.42) | 0.33 | 0.56 |
|  |  | No | 35 (12.92) | 101 (37.27) |  |  |
| **6.** | **Willingness to study the same degree** | Yes | 67 (25.09) | 156 (58.43) | 5.07 | 0.07 |
|  |  | No | 6 (2.25) | 37 (13.86) |  |  |
| **7.** | **Stress during the training at University** | No | 36 (14.63) | 96 (39.02) | 2.14 | 0.54 |
|  |  | Some | 24 (9.76) | 59 (23.98) |  |  |
|  |  | Quite | 5 (2.03) | 25 (10.16) |  |  |
| **8.** | **Employment sector** | Public | 14 (5.19) | 45 (16.67) | 0.42 | 0.80 |
|  |  | Private | 48 (17.78) | 123 (45.56) |  |  |
|  |  | Both | 11 (4.07) | 29 (10.74) |  |  |
| **9.** | **Type of contract** | Permanent | 36 (13.38) | 129 (47.96) | 6.77 | 0.03^**^ |
|  |  | Acting worker | 5 (1.86) | 13 (4.83) |  |  |
|  |  | Temporary | 32 (11.9) | 54 (20.07) |  |  |
| **10.** | **Professional experience** | 0-2 yrs | 17 (6.27) | 20 (7.38) | 9.61 | 0.00^**^ |
|  |  | 3 - 10 yrs | 31 (11.44) | 76 (28.04) |  |  |
|  |  | >10 yrs | 26 (9.59) | 101 (37.27) |  |  |
| **11.** | **Timetable/shift** | Morning | 15 (5.54) | 55 (20.3) | 5.50 | 0.13 |
|  |  | Afternoon | 13 (4.8) | 20 (7.38) |  |  |
|  |  | Morning and afternoon | 37 (13.65) | 108 (39.85) |  |  |
|  |  | Rotating | 9 (3.32) | 14 (5.17) |  |  |
| **12.** | **Adequate training at University** | Yes | - | - | - | - |
|  |  | No | - | - |  |  |
| **13.** | **My job is stressful** | No | 18 (7.26) | 29 (11.69) | 4.38 | 0.11 |
|  |  | Some | 28 (11.29) | 92 (37.1) |  |  |
|  |  | Quite | 19 (7.66) | 62 (25) |  |  |
| ^*^ Percentages are of total respondents in each variable category (% of row totals).  ^**^ Significant (p<0.05)  *χ^2^: Chi-square value*  *p: p-value* | | | | | | |

Table S13. Variables associated with the thought of the physiotherapy as a stressful profession.

| ***Item*** | ***Variables*** | | **My job is stressful*, n(%)^*^*** | | | ***χ^2^*** | ***P*** |
| --- | --- | --- | --- | --- | --- | --- | --- |
|  |  | | **No** | ***Some*** | ***Quite*** |  |  |
| **1.** | **Gender** | Male | 24 (9.68) | 39 (15.73) | 34 (13.71) | 5.30 | 0.07 |
|  |  | Female | 23 (9.27) | 81 (32.66) | 47 (18.95) |  |  |
| **2.** | **Age** | <25 yrs | 2 (0.81) | 16 (6.45) | 21 (8.47) | 20.62 | 0.00^**^ |
|  |  | 26 - 50 yrs | 39 (15.73) | 102 (41.13) | 57 (22.98) |  |  |
|  |  | >50 yrs | 6 (2.42) | 2 (0.81) | 3 (1.21) |  |  |
| **3.** | **Marital status** | Single | 17 (6.85) | 67 (27.02) | 51 (20.56) | 9.02 | 0.06 |
|  |  | Married | 27 (10.89) | 48 (19.35) | 28 (11.29) |  |  |
|  |  | Divorced/Separated | 3 (1.21) | 5 (2.02) | 2 (0.81) |  |  |
| **4.** | **Number of children** | 0 | 21 (8.47) | 70 (28.23) | 61 (24.6) | 20.12 | 0.00^**^ |
|  |  | 1 | 4 (1.61) | 21 (8.47) | 8 (3.23) |  |  |
|  |  | 2+ | 22 (8.87) | 29 (11.69) | 12 (4.84) |  |  |
| **5.** | **Management tasks** | Yes | 25 (10.08) | 59 (23.79) | 41 (16.53) | 0.22 | 0.89 |
|  |  | No | 22 (8.87) | 61 (24.6) | 40 (16.13) |  |  |
| **6.** | **Willingness to study the same degree** | Yes | 40 (16.26) | 105 (42.68) | 59 (23.98) | 10.09 | 0.03^**^ |
|  |  | No | 7 (2.85) | 13 (5.28) | 21 (8.54) |  |  |
| **7.** | **Stress during the training at University** | No | 42 (17.14) | 66 (26.94) | 24 (9.8) | 71.05 | 0.00^**^ |
|  |  | Some | 5 (2.04) | 48 (19.59) | 29 (11.84) |  |  |
|  |  | Quite | 0 (0) | 4 (1.63) | 26 (10.61) |  |  |
| **8.** | **Employment sector** | Public | 11 (4.45) | 22 (8.91) | 15 (6.07) | 5.48 | 0.24 |
|  |  | Private | 24 (9.72) | 79 (31.98) | 56 (22.67) |  |  |
|  |  | Both | 12 (4.86) | 18 (7.29) | 10 (4.05) |  |  |
| **9.** | **Type of contract** | Permanent | 31 (12.55) | 78 (31.58) | 41 (16.6) | 9.31 | 0.05 |
|  |  | Acting worker | 2 (0.81) | 11 (4.45) | 4 (1.62) |  |  |
|  |  | Temporary | 13 (5.26) | 31 (12.55) | 36 (14.57) |  |  |
| **10.** | **Professional experience** | 0-2 yrs | 4 (1.61) | 14 (5.65) | 15 (6.05) | 14.44 | 0.05 |
|  |  | 3 - 10 yrs | 11 (4.44) | 52 (20.97) | 38 (15.32) |  |  |
|  |  | >10 yrs | 32 (12.9) | 54 (21.77) | 28 (11.29) |  |  |
| **11.** | **Timetable/shift** | Morning | 17 (6.85) | 27 (10.89) | 15 (6.05) | 7.67 | 0.26 |
|  |  | Afternoon | 6 (2.42) | 10 (4.03) | 10 (4.03) |  |  |
|  |  | Morning and afternoon | 22 (8.87) | 70 (28.23) | 48 (19.35) |  |  |
|  |  | Rotating | 2 (0.81) | 13 (5.24) | 8 (3.23) |  |  |
| **12.** | **Adequate training at University** | Yes | 18 (7.26) | 28 (11.29) | 19 (7.66) | 4.38 | 0.11 |
|  |  | No | 29 (11.69) | 92 (37.1) | 62 (25) |  |  |
| **13.** | **My job is stressful** | No | - | - | - | - | - |
|  |  | Some | - | - | - |  |  |
|  |  | Quite | - | - | - |  |  |
| ^*^ Percentages are of total respondents in each variable category (% of row totals).  ^**^ Significant (p<0.05)  *χ^2^: Chi-square value*  *p: p-value* | | | | | | | |
